# Telemedicine Intervention Improves ICU Outcomes

**DOI:** 10.1155/2013/456389

**Published:** 2013-01-08

**Authors:** Farid Sadaka, Ashok Palagiri, Steven Trottier, Wendy Deibert, Donna Gudmestad, Steven E. Sommer, Christopher Veremakis

**Affiliations:** Mercy Hospital St. Louis, Saint Louis University, 621 South New Ballas Road, Suite 4006B, St. Louis, MO 63141, USA

## Abstract

Telemedicine for the intensive care unit (Tele-ICU) was founded as a means of delivering the clinical expertise of intensivists located remotely to hospitals with inadequate access to intensive care specialists. This was a retrospective pre- and postintervention study of adult patients admitted to a community hospital ICU. The patients in the preintervention period (*n* = 630) and during the Tele-ICU period (*n* = 2193) were controlled for baseline characteristics, acute physiologic scores (APS), and acute physiologic and health evaluation (APACHE IV) scores. Mean APS scores were 37.1 (SD, 22.8) and 37.7 (SD, 19.4) (*P* = 0.56), and mean APACHE IV scores were 49.7 (SD, 24.8) and 50.4 (SD, 21.0) (*P* = 0.53), respectively. ICU mortality was 7.9% during the preintervention period compared with 3.8% during the Tele-ICU period (odds ratio (OR) = 0.46, 95% confidence interval (CI), 0.32–0.66, *P* < 0.0001). ICU LOS in days was 2.7 (SD, 4.1) compared with 2.2 (SD, 3.4), respectively (hazard ratio (HR) = 1.16, 95% CI, 1.00–1.40, *P* = 0.01). Implementation of Tele-ICU intervention was associated with reduced ICU mortality and ICU LOS. This suggests that there are benefits of a closed Tele-ICU intervention beyond what is provided by daytime bedside physicians.

## 1. Introduction

More than 5.7 million adults are admitted yearly to intensive care units (ICUs) in the United States [1]. Hospital costs for critically ill patients admitted to the ICU are more than $67 billion annually [1]. Mortality rates for these patients average 10–15%, which is equivalent to approximately 540,000 deaths each year [2]. Evidence shows superior clinical outcomes with a dedicated intensivist staffing model [3]; however, 85–90% of US hospitals do not use this model [4]. The Leap Frog Model was created as an effort to reduce preventable medical mistakes. The model recommends that large employers provide more market reinforcement for the quality and safety of health care. The founders realized that they could take “leaps” forward with their employees, retirees, and families by rewarding hospitals that implement significant improvements in quality and safety. The Leapfrog group has provided guidelines for ICUs, which recommend intensivist-led care for all patients in ICUs, recognizing the opportunity to reduce in-hospital mortality. The Leapfrog model suggests that over 53,000 deaths that occur in the ICU could be avoided if the intensivists staffed all urban hospitals' ICUs nationwide [5]. However, currently there is a shortage of bedside intensivists. In addition, with the aging population, it is projected that the need for intensivists will steadily increase while the supply is likely to stay the same, leading to greater intensivist shortages and thus more difficulty meeting the proposed standards of care [4]. As a result, telemedicine for the ICU (Tele-ICU) was founded as a means of delivering clinical expertise of intensivists located remotely to hospitals with inadequate access to intensive care specialists [6]. Tele-ICU intensivists and nurses use audio and video links to assist bedside caregivers in monitoring and managing critically ill patients. Published outcome data describing the effects of Tele-ICU programs have yielded conflicting results. The purpose of this study was to assess the effect of a Tele-ICU 24/7 program on the mortality and length of stay (LOS) of ICU patients using a pre- and post-intervention model in a community-based hospital. 

## 2. Methods

We preformed an unblinded study of Tele-ICU intervention at a large community-based hospital. Telemedicine physicians were located at a central location at Mercy Hospital; however, the community-based hospital monitored by telemedicine is located remotely in another state. For the primary analysis, a representative sample of preintervention cases was obtained by identifying consecutive hospital cases from a data base of all ICU admissions. This investigation was a retrospective study of patients admitted to a 17-bed medical-surgical ICU of a community hospital, between July 2009 and March 2011. Tele-ICU was implemented starting January 1st, 2010. Hospital staff physicians were asked at the time of the Tele-ICU program implementation to indicate their requested level of intervention from the Tele-ICU between level I and level II. As a result, Tele-ICU physicians were automatically consulted on the patients based on the level assigned by hospital staff physician. For level I staff physicians (52.5%), the Teleintensivist could initiate interventions for any urgent/emergent conditions as well as evidence-based therapies and hospital-approved protocols. For level II staff physicians (47.3%), the Teleintensivist had full order writing privileges and fully managed the patients. There was no phase-in (learning period) for this institution, as Tele-ICU has been implemented for other institutions for about a year before this community hospital was added. By the time this hospital was monitored by Tele-ICU, no new learning was needed. Baseline data collected for 6 months before Tele-ICU was compared with data collected for 15 months after Tele-ICU program.

The ICU model of care in the preintervention period was an “Open” ICU where the primary care provider (PCP) admitted patients to the ICU and continues to act as the primary physician, with consult on some patients with an inhouse intensivist who will manage these patients during day time only. The ICU in the intervention period remained “Open.” The only new ICU intervention was by Tele-ICU. The Tele-ICU program operated on a 24/7 schedule and was staffed by two board-certified intensivists, five critical care nurses with at least 5 years of bedside critical care experience (5–35 years, with an average of 18.6 years), and two unit secretaries. Each telemedicine workstation consisted of the following: Philips VISICU *e*Care Manager electronic critical care system, Philips VISICU Smart Alerts, Philips VISICU camera system (Philips, Amsterdam, The Netherlands) in each ICU room allowing two-way voice and two-way video communication in each ICU room, mirrored real-time Philips bedside monitors and PACS (Picture Archiving and Communications System) for Radiology, and a Powerchart for patient data from the hospital. The *e*Care Manager has an outbound link for all notes, physician orders and nursing documentation from *e*Care Manager to patients electronic and paper charts. This study was approved by Mercy Hospital St. Louis Institutional Review Board. Informed consent was waived for this study, since this was a retrospective review of data that pertained no harm to the patients and confidentiality was maintained at all times.

## 3. Statistical Analysis

The study was powered to have an 80% probability for detecting a 3% improvement in ICU LOS using 1 : 4 allocation patients in the Tele-ICU and control groups at a significance level of 0.05. Admission and laboratory values were extracted electronically. The Acute Physiologic Scores (APS) and Acute Physiologic and Chronic Health Evaluation IV (APACHE IV) scores were used to measure severity of illness. Severity-adjusted ICU and hospital mortality were prescribed as the main outcomes. Other outcomes included ICU and hospital LOS. Basic descriptive statistics were calculated for continuous variables and included, presented as the frequency, percentage, or mean +/− standard deviation (SD). Log transformation and geometric mean comparison were preformed when data was distributed nonnormally. Comparison of log transformed means was made using an *F* test. Comparison of categorical data was made using Fisher exact test, *χ*-squared test, or by logistic regression. 

Logistic regression analysis was used to compare the risk of mortality with the presence or absence of Tele-ICU program between the study periods. Comparisons of continuous outcomes were modeled using general linear models. In addition, the same outcome comparisons were made for patients admitted during the day (7 am till 7 pm) and patients admitted at night (7 pm till 7 am). All analyses were two sided and performed on SPSS 20.0 (SPSS Inc., Chicago, IL, USA). 

## 4. Results

### 4.1. Whole Study Population

A total of 2823 adult patients were studied (Table 1). The patients in the preintervention period (*n* = 630) and during the Tele-ICU period (*n* = 2193) were controlled for baseline characteristics, APS, and APACHE IV scores. Mean APS scores were 37.1 (SD, 22.8) and 37.7 (SD, 19.4) (*P* = 0.56), and mean APACHE IV scores were 49.7 (SD, 24.8) and 50.4 (SD, 21.0) (*P* = 0.53), respectively. ICU mortality was 7.9% during the preintervention period compared with 3.8% during the Tele-ICU intervention period (odds ratio (OR) = 0.46, 95% confidence interval (CI), 0.32–0.66, *P* < 0.0001) (Table 2). Hospital mortality was 8.8% compared with 6.9% respectively (OR = 0.76, 95% CI, 0.55–1.0, *P* = 0.1). ICU length of stay (LOS) in days was 2.7 (SD, 4.1) compared with 2.2 (SD, 3.4), respectively (hazard ratio (HR) = 1.16, 95% CI, 1.00–1.40, *P* = 0.01) (Table 2). Hospital LOS in days was 5.2 (SD, 6.1) compared with 6.2 (SD, 7.4), respectively (HR = 1.30, 95% CI, 1.25–1.35, *P* = 0.00) (Table 2).

### 4.2. Study Population Stratified by AM Shift versus PM Shift

Since the ICU under study was staffed by bedside PCPs during the day, we wanted to see if the outcome benefit found above was primarily at nighttime rather than during the day. As a result, we evaluated the outcomes of patients admitted between 7 am and 7 pm (AM shift) separately from patients admitted to the ICU between 7 pm and 7 am (PM shift). There were a total of 1026 patients admitted during AM shift (199 in the preintervention group, and 827 in the Tele-ICU group). ICU mortality was 9.0% during the preintervention period compared with 4.9% during the Tele-ICU intervention period (OR = 0.52, 95% CI, 0.29–0.93, *P* = 0.02) (Table 3). Hospital mortality was 9.5% compared with 9.0%, respectively (OR = 0.94, 95% CI, 0.55–1.6, *P* = 0.8). ICU LOS and Hospital LOS results were similar to the whole study population results (data not shown). There were a total of 1797 patients admitted during PM shift (431 in the preintervention group, and 1366 in the Tele-ICU group). ICU mortality was 7.6% during the preintervention period compared with 3.4% during the Tele-ICU intervention period (OR = 0.42, 95% CI, 0.26–0.66, *P* = 0.0001) (Table 4). Hospital mortality was 8.8% compared with 6.0%, respectively (OR = 0.66, 95% CI, 0.44–0.98, *P* = 0.04). ICU LOS and Hospital LOS results were also similar to the whole study population results (data not shown). 

## 5. Discussion

 This study was designed to assess the impact of Tele-ICU on outcomes. Tele-ICU intervention significantly improved ICU mortality, and ICU LOS. There was a trend towards improvement in hospital mortality; however, it was associated with increased hospital length of stay in this study. This could be because more patients survived in the ICU and thus stayed more days in the hospital. Similar findings were shown whether patients were admitted at night (PM shift) or during the day (AM shift). ICU mortality significantly decreased with Tele-ICU intervention in both PM and AM shift groups. There was a trend towards decrease in hospital mortality in the AM shift group and a statistically significant decrease in hospital mortality in the PM shift group. Hospital LOS increased in both groups, which again could be because more patients survived. Although patients were divided into level I patients (52.5%, the Tele-intensivist could initiate interventions for any urgent/emergent conditions as well as evidence-based therapies and hospital-approved protocols) and level II patients (47.3%, the Tele-intensivist had full-order writing privileges and fully managed the patient), similar findings to the above were found for the two groups (data not shown). The overall conclusion of this analysis supports the claim that Tele-ICU can improve ICU survival, hospital survival, and shorten ICU LOS. 

Our findings are similar to other studies on Tele-ICU. Breslow et al. demonstrated lower hospital mortality for ICU patients during the period of remote ICU care (9.4% versus 12.9%; relative risk 0/73; 95% CI, 0.55–0.95) and shorter ICU LOS (3.63 days; 95% CI, 3.21–4.04 versus 4.35 days; 95% CI 3.93–4.78) [7]. Rosenfeld et al. reported a decrease in severity-adjusted ICU mortality by 45% and a decrease in hospital mortality by 30% [8]. McCambridge et al. reported 29.5% reduction in hospital mortality with Tele-ICU [9]. The New England Healthcare Institute showed that ICU mortality decreased by more than 20% and hospital mortality decreased by 13% [10]. ICU LOS decreased by an average of almost 2 days or 30%. A study by Zawada et al. showed that Tele-ICU was associated with a reduction in severity-adjusted ICU mortality (OR = 0.35; *P* = 0.007), decreased ICU LOS (3.79 versus 2.08 days; *P* = 0.001), and reduced hospital LOS (10.08 versus 7.81 days; *P* = 0.001) [11]. Lilly et al. looked at the association of a Tele-ICU intervention with hospital mortality, LOS, and complications that are preventable by adherence to best practices. They reported a hospital mortality rate of 13.6% (95% CI, 11.9% to 15.4%) during the preintervention period compared with 11.8% (95% CI, 10.9% to 12.8%) during the Tele-ICU intervention period and hospital LOSs of 9.8 days and 13.3 days, respectively [12]. Most recently, Willmitch et al. found significant decreases in severity-adjusted hospital LOS of 14.2%, ICU LOS of 12.6%, and relative risk of hospital mortality of 23% with Tele-ICU intervention [13]. In the current study, however, the major outcome benefit was observed in ICU mortality and LOS rather than hospital outcomes. It is not likely that a Tele-ICU program would impact hospital outcomes but does raise the question as to whether there is potential benefit of telemedicine if applied to hospital ward patients.

Our study has several limitations. This was a retrospective study and thus reports associations rather than cause and effect relationships. The fact that this was a single medical center study should be taken into account when considering the results of this study. The results may not be generalized to other institutions. However, because the design of the study included a heterogeneous ICU population, it may better reflect the outcomes that could be achieved in an actual clinical practice rather than those observed in randomized controlled trials. Another limitation is the potential difference in number of cases with “do not resuscitate” status and life withdrawal cases between the 2 groups; these were not reported in our study. In addition, the study did not evaluate the effect of Tele-ICU on compliance with critical care processes before intervention such as deep vein thrombosis, stress ulcer prophylaxis, ventilator associated pneumonia, ventilator-free days, and catheter-related blood stream infections. The data from the Tele-ICU period revealed compliance of almost 100% with these processes. Another limitation of this study is that it was not possible to account for temporal changes that occur over time and may influence the outcome. The current study represents a large number of patients (2823) and its findings are similar to other studies on Tele-ICU, both of which are points of strength. Strength of this study was the ability to control for important markers of severity of illness (APS and APACHE IV) helping to validate the results. A strong association of Tele-ICU interventions for patients admitted during the nighttime than those admitted during the daytime for ICU mortality and LOS was found in our study, suggesting that intensivist involvement in off-hour's cases was an important contributor to the association of the intervention with improved outcomes. Studies reporting higher mortality for ICU patients admitted at night may suggest that part of the lower mortality and shorter length of stay may be due to the fact that a well-rested on duty intensivist was assisting in the management of the patients at night using Tele-ICU workstations and tools [14].

The deployment of a Tele-ICU program is a complex process consisting of hundreds of discrete elements and the introduction of a new culture for management of ICU patients. In addition, building relationships and fostering acceptance of a Tele-ICU program by bedside nurses, private practice physicians, and bedside intensivists take time and patience. Our Tele-ICU physicians and nurses were instructed to be as proactive as possible. In addition, dozens of protocols have been instituted by collaboration among the Tele-ICU team and the bedside team of administrators, physicians, and nurses. A major reason why some studies failed to detect significant associations between Tele-ICU interventions and outcome is the low rates of collaboration among Tele-ICU and bedside physicians and nurses (34%–36%) [15, 16]. We attest from our own experience that bedside and Tele-ICU team collaboration is an important determinate of favorable outcomes resulting from Tele-ICU intervention. 

What makes us unique is our approach to Tele-ICU with two board-certified intensivists and five highly experienced critical care nurses heavily involved in patient care 24 hours/day. The intensivists worked 12-hour shifts and were thus well rested for the shift. In addition, our approach excelled in building relationships and fostering acceptance of a Tele-ICU program by bedside nurses, private practice physicians, and bedside intensivists in the monitored facility. We believe that all the above mentioned are necessary for the outcome benefits from any remote tele-medicine intervention to be implemented.

## 6. Conclusion

This retrospective pre- and post-Tele-ICU study documented statistically significant decreases of severity of illness-adjusted mortality and length of stay in cases admitted to an adult ICU in a single ICU of a community hospital.

## Figures and Tables

**Table 1:** General characteristics of study patients.

	Preintervention (*n* = 630)	Tele-ICU (*n* = 2193)	*P* value
Patient characteristics			
Age in yrs, mean, SD	66.1 (14.5)	67.1 (15.3)	0.1
Male, *n*, %	334 (53)	1162 (53)	0.8
APS^a^, mean, SD	37.1 (22.8)	37.7 (19.4)	0.6
APACHE^b^ IV, mean, SD	49.7 (24.8)	50.4 (21.0)	0.5
Admission diagnosis by organ system, *n*, %			
Cardiovascular	369 (58.6)	1202 (54.8)	0.1
Pulmonary	69 (10.9)	299 (13.6)	0.1
Gastrointestinal	38 (6.0)	175 (8.0)	0.1
Neurologic	24 (3.8)	108 (4.9)	0.2
Genitourinary/renal	85 (13.5)	240 (10.9)	0.1
Endocrinologic	38 (6.0)	149 (6.8)	0.2
Hematologic	7 (1.2)	20 (1.0)	0.8

^a^APS: acute physiologic score.

^b^APACHE: Acute Physiologic and Chronic Health Evaluation.

**Table 2:** Mortality and length of stay outcomes in whole study population.

Outcome	Preintervention (*n* = 630)	Tele-ICU (*n* = 2193)	Effect estimate	*P* value
ICU mortality, *n*, %	50 (7.9)	84 (3.8)	0.46 ( 0.32–0.66)^a^	<0.01
Hospital mortality, *n*, %	56 (8.8)	153 (6.9)	0.76 (0.55–1.0)^a^	0.1
ICU length of stay, mean (SD) median [IQR], *d *	2.7 (4.1) 1.6 (0.9–2.5)	2.2 (3.4) 1.0 (1.0-2.0)	1.16 (1.00–1.40)^b^	0.01
Hospital length of stay, mean (SD) median [IQR], *d *	5.2 (6.1) 3.6 (1.5–6.1)	6.2 (7.4) 4.2 (2.1–2.8)	1.30 (1.25–1.35)^b^	0.00

Abbreviations: ICU: intensive care unit; IQR: interquartile range.

^a^Indicates odds ratio (95% confidence interval).

^b^Indicates hazard ratio ( 95% confidence interval).

**Table 3:** Mortality outcomes in patients admitted to ICU in AM (7 am–7 pm).

Outcome	Preintervention (*n* = 199)	Tele-ICU (*n* = 827)	Effect estimate	*P* value
ICU mortality, *n*, %	18 (9.0)	41 (4.9)	0.52 (0.29–0.93)^a^	0.02
Hospital mortality, *n*, %	19 (9.5)	75 (9.0)	0.94 (0.55–1.60)^a^	0.8

^a^Indicates odds ratio (95% confidence interval).

**Table 4:** Mortality outcomes in patients admitted to ICU in PM (7 pm–7 am).

Outcome	Preintervention (*n* = 431)	Tele-ICU (*n* = 1366)	Effect estimate	*P* value
ICU mortality, *n*, %	33 (7.6)	46 (3.4)	0.42 (0.26–0.66)^a^	0.0001
Hospital mortality, *n*, %	38 (8.8)	82 (6.0)	0.66 (0.44–0.98)^a^	0.04

^a^Indicates odds ratio (95% confidence interval).
